# Construction of Opa-Positive and Opa-Negative Strains of *Neisseria meningitidis* to Evaluate a Novel Meningococcal Vaccine

**DOI:** 10.1371/journal.pone.0051045

**Published:** 2012-12-12

**Authors:** Manish Sadarangani, J. Claire Hoe, Martin J. Callaghan, Claire Jones, Hannah Chan, Katherine Makepeace, Hélène Daniels-Treffandier, Mary E. Deadman, Christopher Bayliss, Ian Feavers, Peter van der Ley, Andrew J. Pollard

**Affiliations:** 1 Department of Paediatrics, University of Oxford and the NIHR Oxford Biomedical Research Centre, Oxford, United Kingdom; 2 Division of Infectious Diseases, Department of Pediatrics, University of British Columbia and BC Children's Hospital, Vancouver, British Columbia, Canada; 3 Division of Bacteriology, National Institute for Biological Standards and Control, Potters Bar, Hertfordshire, United Kingdom; 4 Department of Genetics, University of Leicester, Leicester, United Kingdom; 5 Department of Vaccinology, National Institute of Public Health and the Environment (RIVM), Bilthoven, The Netherlands; University of Würzburg, Germany

## Abstract

*Neisseria meningitidis* is a major global pathogen causing invasive disease with a mortality of 5–10%. Most disease in developed countries is caused by serogroup B infection, against which there is no universal vaccine. Opacity-associated adhesin (Opa) proteins are major meningococcal outer membrane proteins, which have shown recent promise as a potential novel vaccine. Immunisation of mice with different Opa variants elicited high levels of meningococcal-specific bactericidal antibodies, demonstrating proof in principle for this approach. Opa proteins are critical in meningococcal pathogenesis, mediating bacterial adherence to host cells, and modulating human cellular immunity via interactions with T cells and neutrophils, although there are conflicting data regarding their effects on CD4^+^ T cells. We constructed Opa-positive and Opa-negative meningococcal strains to allow further evaluation of Opa as a vaccine component. All four *opa* genes from *N. meningitidis* strain H44/76 were sequentially disrupted to construct all possible combinations of *N. meningitidis* strains deficient in one, two, three, or all four *opa* genes. The transformations demonstrated that homologous recombination of exogenous DNA into the meningococcal chromosome can occur with as little as 80 bp, and that minor sequence differences are permissible. Anti-Opa bactericidal antibody responses following immunisation of mice with recombinant Opa were specific to the Opa variant used in immunisation. No immunomodulatory effects were observed when Opa was contained within meningococcal outer membrane vesicles (OMVs), compared to Opa-negative OMVs. These observations support the incorporation of Opa in meningococcal vaccines.

## Introduction

*Neisseria meningitidis* causes up to 500,000 cases of meningitis and septicaemia worldwide annually, with a mortality rate of approximately 10% [1]. Most cases of disease are caused by 5 of the 13 meningococcal serogroups: A, B, C, Y and W135. Protein-polysaccharide conjugate vaccines are now available for all of these serogroups except serogroup B, since epitopes of this polysaccharide capsule are cross-reactive with the human neural cell adhesion molecule [2], and it is therefore not immunogenic in humans [3]. Serogroup B organisms are currently the major cause of disease in most temperate countries [4], [5], [6], [7], [8]. A number of vaccines based on different combinations of subcapsular antigens are currently in development for the prevention of serogroup B disease [9], including different outer membrane vesicle (OMV) vaccines using genetically modified meningococci [9], [10], [11], [12].

The Opacity-associated (Opa) adhesin proteins are some of the major proteins found in the outer membrane of *N. meningitidis* and *N. gonorrhoeae*, which can express up to four or eleven different Opa variants respectively, encoded at loci dispersed throughout the genome [13], [14], [15]. There are at least 338 allelic variants (www.neisseria.org, accessed 17th April 2012) due to sequence variability in three of the four surface-exposed loops [16], [17]. Opa proteins play a critical role in meningococcal pathogenesis by mediating bacterial adherence to the nasopharynx and modulating human cellular immunity via interactions with T cells and neutrophils [18], [19]. Opa proteins are a potential novel vaccine candidate for the prevention of meningococcal disease caused by all serogroups, and have the potential to protect against the hyperinvasive isolates which are responsible for most of the disease burden [20], [21]. Bactericidal antibodies are currently accepted as the correlate of protection against meningococcal disease, and anti-Opa bactericidal antibodies have been demonstrated in patients following infection with *N. meningitidis* and in recipients of serogroup B OMV vaccines [22], [23], [24], [25], [26], [27], [28], [29]. Immunisation of mice with recombinant Opa proteins or Opa-containing liposomes has also elicited the production of high levels of bactericidal antibodies [21], [30]. One obstacle to human trials of an Opa vaccine is an observation that these proteins might inhibit CD4^+^ T cell proliferation under certain conditions *in vitro*, although there are conflicting published data about the validity of this observation [31], [32], [33], [34], [35]. The potential expression of multiple Opa proteins from any given isolate has made it difficult to study the immunoprotective or immunomodulatory effects of specific Opa proteins expressed on the bacterial surface.

In order to individually assess different Opa variants as potential vaccine candidates, we constructed Opa-positive and Opa-negative meningococcal strains, from the same parent strain, expressing different Opa proteins. A library of strains was derived from *N. meningitidis* strain H44/76 in which one, two, three or four *opa* genes had been disrupted, for further evaluation of Opa proteins as a potential meningococcal vaccine candidate. These strains were utilised to examine the specificity of the anti-Opa response following immunisation of mice with recombinant Opa protein and Opa-positive or Opa-negative OMVs.

## Results

### Construction of Δopa plasmids

**Figure 1:**
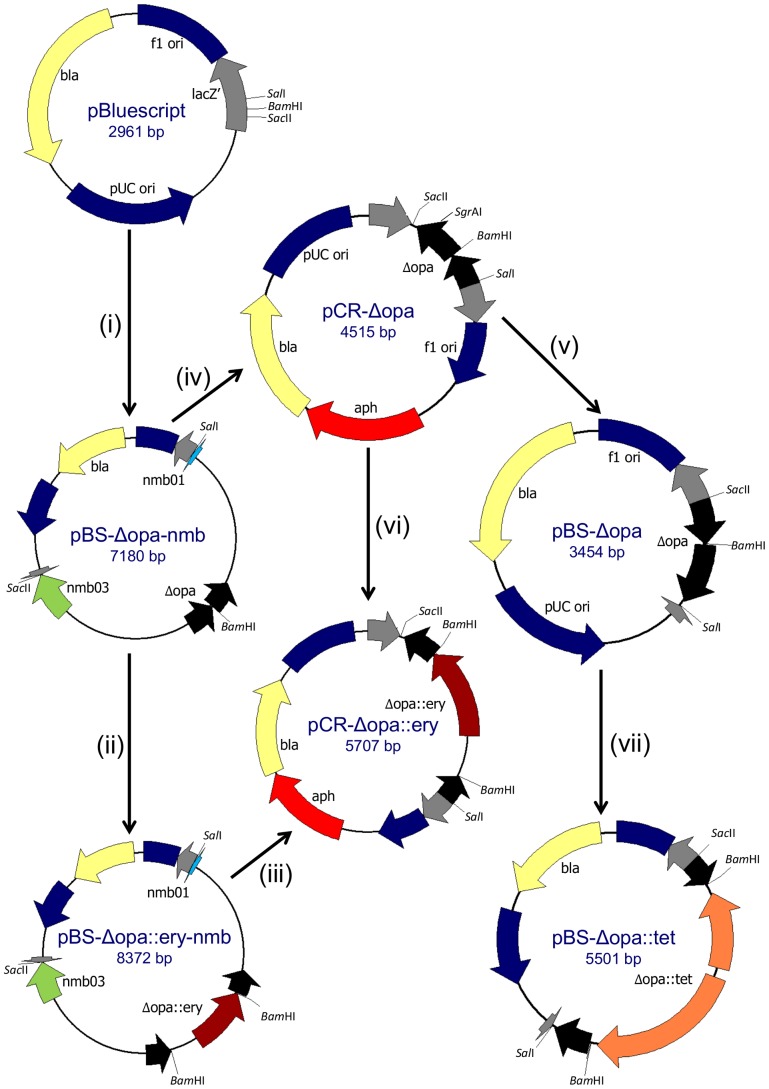
Summary of cloning steps in construction of *Δopa* plasmids. A general scheme is depicted. Different steps were used for each *opa* gene, as described below and in the text. (i) The 5′ and 3′ ends of *opaA, opaD or opaJ* (black) were amplified by PCR (table 1) along with the adjacent locus-specific genes, depicted as *nmb01* (blue) and *nmb03* (green). Novel *Sal*I and *Bam*HI or *Bam*HI and *Sac*II restriction sites were introduced at the ends of the amplicons. Each PCR product was then cloned separately into pCR2.1-TOPO (not shown). The two ends of each *opa* were excised from the pCR2.1 plasmids and cloned sequentially into pBluescript, resulting in locus-specific plasmids pBS-ΔopaA-nmb, pBS-ΔopaD-nmb and pBS-ΔopaJ-nmb. These plasmids therefore contained a modified *opa* gene, *Δopa*, which contained a 185 bp deletion. (ii) A 1,192 bp *Bam*HI fragment carrying *ermC* was cloned into pBS-ΔopaJ-nmb to produce pBS-ΔopaJ::ery-nmb. (iii) *ΔopaJ::ery* was amplified from pBS-ΔopaJ::ery-nmb using primers OpaFSalI and OpaRSacII (table 1), excluding *opaJ* locus-specific regions. The resulting amplicon was cloned into pCR2.1-TOPO to generate the generic plasmid pCR-Δopa::ery. (iv) *ΔopaA* and *ΔopaD* were amplified from their respective pBS-Δopa-nmb plasmids using primers OpaFSalI and OpaRSacII. Each modified *opa* was cloned into pCR2.1-TOPO, resulting in the generic plasmids pCR-ΔopaA and pCR-ΔopaD. (v) *ΔopaA* and *ΔopaD* were excised from the pCR2.1 plasmids by double digestion with *Sal*I and *Sac*II and cloned into pBluescript which had been similarly prepared, to create pBS-Δopa plasmids. (vi) A 1,194 bp *Sgr*AI fragment carrying *ermC* was cloned into pCR-ΔopaD to produce pBS-ΔopaD::ery. (vii) A kanamycin (KanR) or tetracycline (TetR) resistance cassette was introduced into pBS-ΔopaD, or KanR was introduced into pBS-ΔopaA, resulting in generic *Δopa* plasmids containing selectable markers.

Locus-specific *Δopa* plasmids were designed to facilitate sequential, targeted disruption of the four *opa* genes (*opaA*, *opaB*, *opaD* and *opaJ*) of *N. meningitidis* strain H44/76. These *Δopa* plasmids each contained a disrupted *opa* gene flanked by upstream and downstream sequences specific for the relevant *opa* locus, with or without an antibiotic resistance cassette (for selection following transformation) (figure 1). However, some of the cloning steps were unsuccessful; it was not possible to construct locus-specific *ΔopaB* plasmids, and insertion of an antibiotic resistance cassette was only possible for the *ΔopaJ* plasmid. An alternative strategy was devised based on the finding that the four *opa* genes of strain H44/76 possess 96% sequence identity for the 253 bp at the 5′ end and 93% for the 228 bp at the 3′ end, with 99% similarity between *opaA* and *opaJ*, and 97% between *opaB* and *opaD* within these regions. Generic *Δopa* plasmids were therefore constructed, without the flanking locus-specific regions, to enable non-specific disruption of *opa* genes (figure 1). Locus-specific plasmids all included the suffix -nmb. PCR primers are listed in table 1.

**Table 1:** PCR primers used for amplification of *opa* genes from *N. meningitidis* and construction of *Δopa* plasmids.

Target gene(s)	Forward primer	Reverse primer	Product size (bp)
*opaA - nmb0441*	nitF1SalI§(5′-GCACGTGTCGACACAGCATGATTGTCGATCC-3′)	083BamHI¶(5′-CTATATGGATCCGCGCGTCGCCTACGGAC-3′)	511
*opaA - nmb0444* *	085-MSBamHI¶(5′-TTTTCTGGATCCGGCATAATCTGCCGCTATTC-3′)	NMB0444-4SacII§(5′-TGAAGCCCGCGGGTCAGCACATAGTTGACG-3′)	2838
*opaB - nmb1634* †	NMB1634-4SalI§(5′-GCCGTAGTCGACTTCTTCCGATCCCAACC-3′)	085BamHI¶(5′-TTTTCTGGATCCGGCATAATCTGCCGCTATCC-3′)	2556
*opaB - nmb1637*	083BamHI¶(5′-CTATATGGATCCGCGCGTCGCCTACGGAC-3′)	0464 opaBrevSacII∥(5′-TTACCGCCGCGGAAGGCGAGGTAGGATTGC-3′)	941
*opaD - nmb1464*	NMB1464-3SalI§(5′-CAAAAGGTCGACTGCCAAAGCCTGAGATTGC-3′)	083BamHI¶(5′-CTATATGGATCCGCGCGTCGCCTACGGAC-3′)	1559
*opaD - ppx* ‡	085BamHI¶(5′-TTTTCTGGATCCGGCATAATCTGCCGCTATCC-3′)	opaDrevdSacII§(5′-TTTCGACCGCGGAGGCGGAATGCTTGTGATAG-3′)	2671
*opaJ - nmb0925*	acthR2SalI§(5′-GCGACGGTCGACAGGAGCAGTTCGCCTTGAG-3′)	083BamHI¶(5′-CTATATGGATCCGCGCGTCGCCTACGGAC-3′)	1968
*opaJ - pip*	085-MSBamHI¶(5′-TTTTCTGGATCCGGCATAATCTGCCGCTATTC-3′)	pipSEQRSacII**(5′-CCGGTTCCGCGGATTTTCAGCAATCGGCGCG-3′)	2360
*ermC*(EryR)††	ery-bamf§(5′-GATCCCGGATCCTGCAGGAATTCGATATCAAGC-3′)	ery-bamr§(5′-CCGGGCGGATCCTCGAGGTCGACGGTATCG-3′)	1210
*ermC*(EryR)††	ery-sgrf§(5′-TGGATCCACCGGTGTGCAGGAATTCGATATCAAGC-3′)	ery-sgrr§(5′- TACCGGCACCGGCGTCGAGGTCGACGGTATCG-3′)	1214
*tetA – tetR*(TetR)***	NmnDUS5§ ‡‡(5′-GGACGATCG *ATGCCGTCTGAA*ACCAATACAATGTAGGCTGC-3′)	NmnDUS3§ ‡‡(5′-GGACGATCGT*TTCAGACGGCAT*CGAAAAACCTAAAAGAGC-3′)	1990
*Δopa* *Δopa::eryR*	OpaFSalI§(5′ TTCCGCGTCGACGGCGGCAAGTGAAGACG-3′)	OpaRSacII§(5′ ATGCCGCCGCGGGGTTCAGACGGCATCG-3′)	5841776∥∥

Restriction sites within primer sequences are underlined. *nmb* nomenclature as defined in the published sequence of *N. meningitidis* strain MC58 (GenBank accession number AE002098), which was used to design primers in this study since the sequence of H44/76 had not been published.

* *nmb0443* is adjacent to *opaA* but no unique primer site could be identified within *nmb0443* so *nmb0444* was used;

† *nmb1635* is adjacent to *opaB* but the putative coding sequence is only 222 bp so *nmb1634* was used;

‡ *nmb1466* is adjacent to *opaD* but primers within *ppx* have been published following successful use;

§ designed during this study;

¶ from Hobbs *et al.* [75];

∥ from Morelli *et al.* [76];

** from Maiden *et al.* [77];

†† EryR = erythromycin resistance cassette, which contains the gene *ermC*;

‡‡ DNA uptake sequences within NmnDUS5 and NmnDUS3 are italicised;

*** TetR = tetracycline resistance cassette, which contains the genes *tetA* and *tetR*;

∥∥ product was 584 bp when amplifying *Δopa* and 1776 bp when amplifying *Δopa::ery*.

### ΔopaJ plasmids

The 5′ and 3′ ends of *opaJ* were amplified separately along with adjacent sections of the neighbouring genes *nmb0925* and *pip*, introducing *Sal*I and *Bam*HI or *Sac*II and *Bam*HI restriction sites at the ends of the amplicons. These were cloned separately into pCR2.1-TOPO before being excised with the relevant restriction enzymes and ligated together in the plasmid vector pBluescript II (SK-) to construct pBS-ΔopaJ-nmb. An erythromycin resistance cassette (EryR) containing *ermC* was amplified from the plasmid pER2 [36], using primers ery-bamf and ery-bamr to introduce *Bam*HI sites flanking *ermC*. The resulting amplicon was cloned into pCR2.1-TOPO, excised with *Bam*HI and inserted into the *Bam*HI site of pBS-ΔopaJ-nmb, resulting in pBS-ΔopaJ::ery-nmb. *ΔopaJ::ery* was amplified from this plasmid using primers OpaFSalI and OpaRSacII to exclude adjacent locus-specific genes. This amplicon contained Δ*opaJ::ery* and the immediately adjacent *opa* homologous regions, including a downstream DNA uptake sequence (DUS), and novel *Sal*I and *Sac*II sites at either end. This was cloned into pCR2.1-TOPO to construct pCR-opaJ::ery.

### ΔopaD plasmids

pBS-opaD-nmb was constructed in a similar fashion to pBS-opaJ-nmb. Δ*opaD* was amplified from this plasmid using OpaFSalI and OpaRSacII and cloned into pCR2.1-TOPO, resulting in pCR-ΔopaD. *ΔopaD* was excised using *Sal*I and *Sac*II and ligated into pBluescript, which had been similarly prepared, to create pBS-ΔopaD. EryR was inserted into pCR-ΔopaD by amplifying *ermC* from pER2 using primers ery-sgrf and ery-sgrr, to introduce *Sgr*AI sites flanking *ermC*, before cloning it into the *Sgr*AI site within *opaD*, to obtain pCR-ΔopaD::ery. A kanamycin resistance cassette (KanR) was excised from pUC4-kan (GenBank accession number X06404) using *Bam*HI. A tetracycline resistance cassette (TetR) was constructed by excising a *Hind*III-*Sal*I fragment from pHVT1 [37], which was cloned into a plic2A vector and amplified by PCR using primers NmnDUS5 and NmnDUS3 (table 1). This reduced the size of TetR by several hundred base pairs, and introduced novel *Pst*I sites and neisserial DNA uptake sequences. This amplicon was cloned into a TA vector, excised with *Pst*I and inserted into pUC4-kan which had been similarly prepared, thus replacing KanR with TetR to produce pUC4NmDUS. pBS-ΔopaD::kan and pBS-ΔopaD::tet were constructed by insertion of either KanR or TetR, respectively, into the *Bam*HI site of pBS-ΔopaD.

### ΔopaA plasmids

pBS-ΔopaA-nmb was constructed as described for pBS-ΔopaJ-nmb. pBS-ΔopaA and pBS-ΔopaA::kan were derived in a similar fashion to the equivalent *ΔopaD* plasmids.

### Construction of opa-deficient meningococci

**Figure 2:**
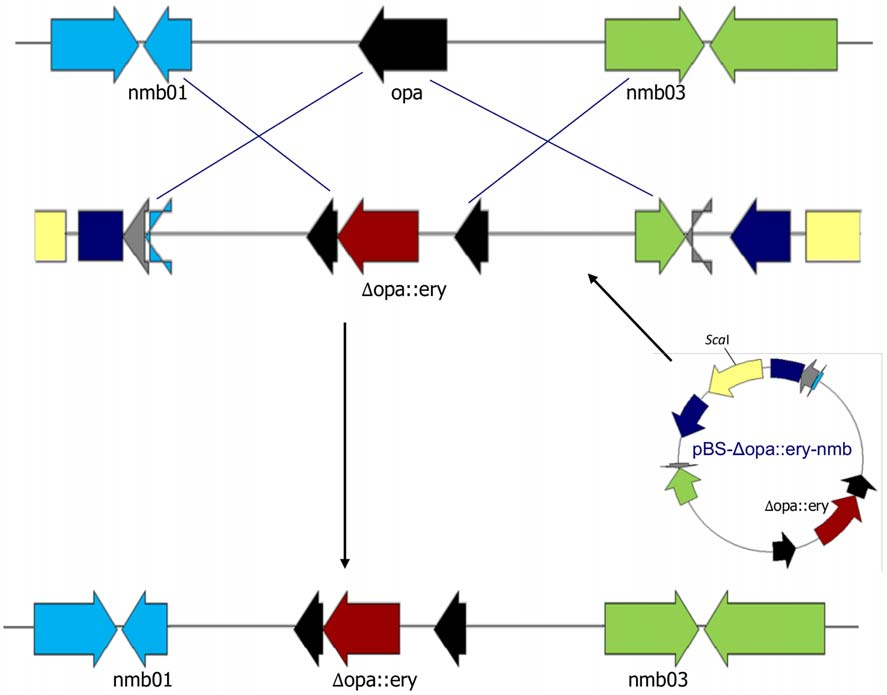
Homologous recombination in *N. meningitidis*. The plasmid pBS-Δopa::ery-nmb contains sequences identical to the 5′ and 3′ ends of *opa* and adjacent locus-specific sequences flanking an erythromycin resistance cassette, EryR. Crossover events between chromosomal and plasmid DNA are illustrated using linearised plasmid. A double crossover event between homologous regions on the plasmid and chromosome allow EryR to be stably inserted into the chromosome at the *opa* locus. Generic plasmids containing *Δopa* without locus-specific regions are able to target multiple *opa* genes for homologous recombination.

**Figure 3:**
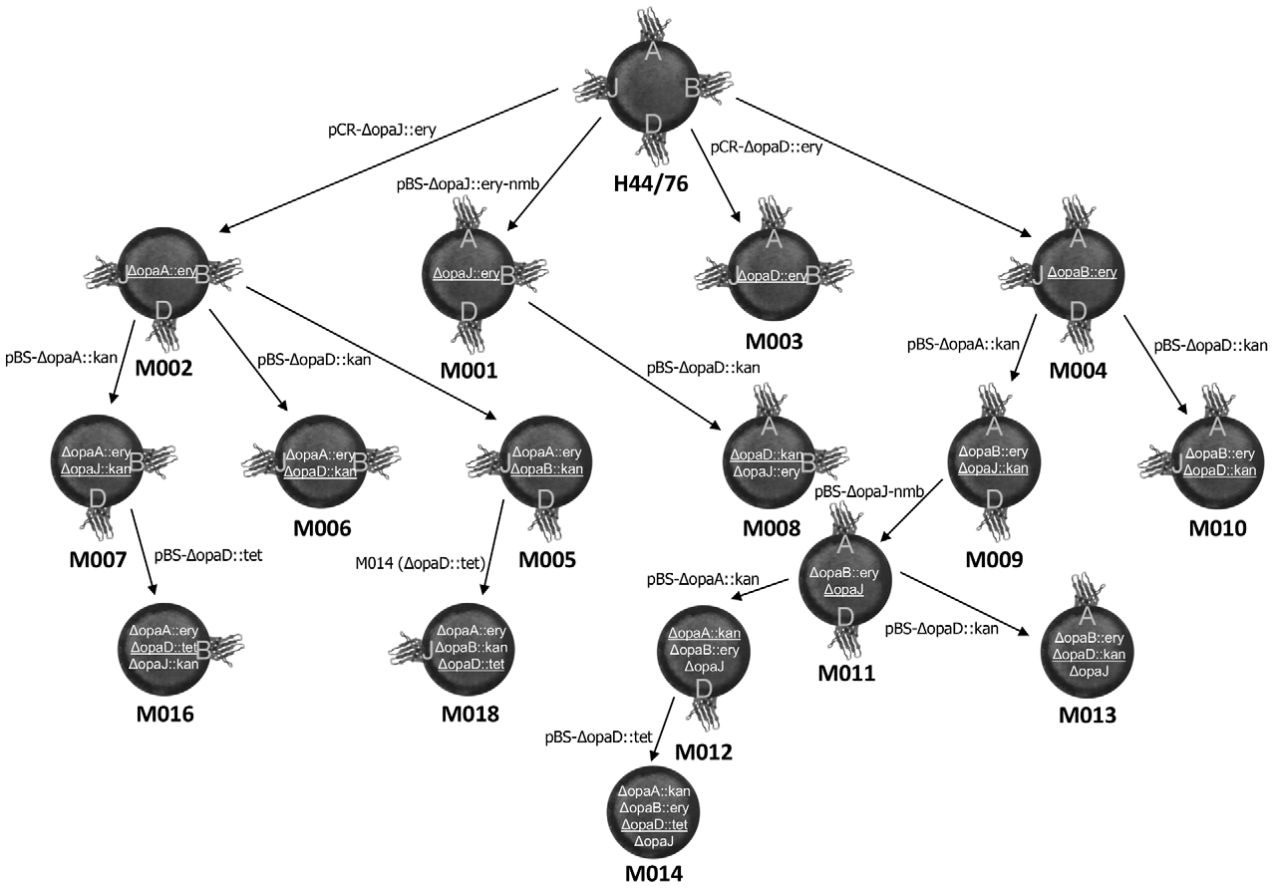
Summary of Opa-deficient mutant meningococci constructed from parent strain H44/76. The plasmid used for each transformation and possible Opa expression of each new strain is indicated, as well as the gene disruptions that have been introduced. For each strain the underlined disruption is the one introduced by the most recent transformation. All possible *opa* combinations were created, including four single *opa*-deficient strains, six double *opa*-deficient strains, four triple *opa*-deficient strains, and an *opa*-negative strain.

*N. meningitidis* strain H44/76 was sequentially transformed with different *Δopa* plasmids to create a library of 15 new *opa*-deficient strains, each theoretically able to express different combinations of the four Opa proteins (figure 2 and figure 3). Plasmids containing different antibiotic resistance cassettes were used to enable selection after each disruption. Plasmids containing Δ*opaA* or Δ*opaJ* were able to target both of these genes and those containing Δ*opaD* disrupted *opaB* and *opaD*. To disrupt all four *opa* genes with only three selectable markers, one of the antibiotic resistance cassettes had to be removed to be re-introduced at a different *opa* locus. Strain M011 was obtained by transformation of M009 using pBS-ΔopaJ-nmb to replace *ΔopaJ::kan* with *ΔopaJ*. A single transformed clone was obtained after screening 10,000 colonies following transformation. This strain was then used to produce two additional strains with KanR introduced within *opaA* or *opaD* and in which *opaJ* remained disrupted. KanR only conferred kanamycin resistance in *N. meningitidis* if inserted into *opa* in the same orientation as the *opa* gene.

pBS-ΔopaA::kan resulted in integration of *ΔopaA::kan* into the *opaA* locus on 70/73 (96%) occasions, and into *opaJ* following other transformations. Similarly, pCR-ΔopaJ::ery only targeted *opaJ* and *opaA*, disrupting *opaJ* 53/68 times (78%). A total of 81 clones were identified where generic plasmids based on *opaD* had undergone homologous recombination. This occurred at the *opaD* locus on 67 occasions (83%), at the *opaB* locus 10 times (12%), and at the *opaA* locus in four clones (5%). Four of the events where integration occurred into the *opaD* locus were single crossover events, with both KanR and TetR being present at the same locus. Although this has been described [38], [39], it is not thought to be a common occurrence in *N. meningitidis*. All other successful transformations were confirmed by PCR to be double crossover events. Transformation rates varied between 10^−5^ and 10^−7^, consistent with previous studies [39], [40].

### DNA sequence analysis of Δopa genes

Nucleotide sequence analysis was performed on multiple loci where recombination occurred between two different *opa* genes, one located on the plasmid and one on the chromosome. Crossover events were observed throughout the homologous flanking regions, including within the first 81 bp of homology, close to the site of the primer used to amplify the *opa* genes, and also within 80 bp of the antibiotic resistance cassette (figure S1). Data from *opa* pentanucleotide coding repeat (CR) sequences, contained within the 5′ end of the Opa open reading frame, revealed that the length of the CR tracts had altered in some *opa* genes during construction of the strain library (table S1). Based on the length of the CR tract, the mature Opa protein should only be expressed on the bacterial surface if the number of CR sequences is a multiple of 3. Therefore, the wild type strain used in this study is not expected to express any Opa proteins, whereas M001 should express both OpaA and OpaD. Only OpaD is predicted to be expressed by M002, M005 and M007. None of the other strains are expected to express any Opa proteins, based on the nucleotide sequence, although they would have the capacity for differential expression of Opa by phase variation (PV) at *opa* loci which had not been disrupted.

### Characterisation of Opa-positive and Opa-negative meningococci

H44/76, M001, M002 and M014 were characterised further and used to provide additional information on the immunogenicity of Opa in the mouse model. Immunodot-blotting of ethanol-fixed bacteria using mAbs 15-1-P5.5 and MN20E12.70 confirmed the expected Opa phenotype based on DNA sequencing data, with expression of OpaD for M001 and M002 and OpaA from M001 only (figure S2). SDS-PAGE and immunoblotting of OMVs produced from these strains confirmed Opa expression consistent with the results of DNA sequence analysis and immunodot-blotting (figure S2). Protein profiles of other major surface-expressed proteins were similar between strains, and based on immunoblotting with specific monoclonal antibodies there was no effect on the expression of PorA, PorB, RmpM or factor H binding protein (fHbp) following manipulation of Opa expression (figure S3).

### Bactericidal antibody responses following immunisation of mice with recombinant Opa proteins and Opa-positive and Opa-negative OMVs

The immunogenicity of recombinant forms of OpaA and OpaD from H44/76 were tested using the selected Opa-positive and Opa-negative strains as target strains in the serum bactericidal antibody (SBA) assay. The recombinant proteins resulted in titres of 1∶256 when the target strain expressed the same Opa variant used for immunisation (table 2). SBA titres of <1∶4 were observed when the target strain in the SBA assay did not express the Opa variant used for immunisation. Immunisation of mice with any of the four OMVs elicited a bactericidal response against all four target strains, demonstrating little difference between the SBA titres (table 2), with a titre of 1∶2048 for most sera against the majority of target strains.

**Table 2:** Serum bactericidal antibody titres of pooled murine sera against 4 target strains, following immunisation with recombinant OpaA and OpaD, and Opa-positive and Opa-negative OMVs.

Antigen used for immunisation		Target strain in SBA assay (Opa phenotype)			
		H44/76 (Opa−)	M014 (Opa−)	M002 (OpaD+)	M001 (OpaA+ OpaD+)
Recombinant protein	**OpaA**	<1∶4	<1∶4	<1∶4	1∶256
	**OpaD**	<1∶4	<1∶4	1∶256	1∶256
**OMV**(Opa phenotype)	**H44/76**(Opa−)	1∶4096	1∶2048	1∶2048	1∶2048
	**M014**(Opa−)	1∶2048	1∶2048	1∶2048	1∶1024
	**M002**(OpaD+)	1∶2048	1∶2048	1∶2048	1∶2048
	**M001**(OpaA+ OpaD+)	1∶2048	1∶1024	1∶2048	1∶2048

Titres represent highest dilution at which there was 50% bacterial survival. Immunisation with recombinant Opa elicited bactericidal antibodies in mice if the same Opa variant was expressed by the target strain in the SBA assay, with a titre of 1∶256 in all cases. Immunisation with any of the OMVs elicited high levels of bactericidal antibodies in mice against all strains in the SBA assay, with titres between 1∶1024 and 1∶4096.

## Discussion

This is the first description of the construction of a library of *opa*-deficient meningococci from a single parent strain, enabling further evaluation of Opa proteins as a potential novel meningococcal vaccine, including investigation of their immunomodulatory effects. This study has also demonstrated significant, specific bactericidal anti-Opa responses following murine immunisation, supporting the pursuit of Opa as a potential vaccine candidate. These strains will be a valuable tool in the assessment of the role of Opa proteins in the pathogenesis of neisserial infection. They will also allow investigation of different Opa variants from the same parent strain to identify any similarities and differences in their functions, and investigate their underlying molecular basis.

Sera from mice immunised with OpaA or OpaD from strain H44/76 were only bactericidal when the same Opa was expressed on the surface of the target strain in the SBA assay. Although previous studies have demonstrated that Opa proteins elicit bactericidal antibodies in mice, the Opa-positive and Opa-negative strains constructed in this study enabled an accurate assessment of specificity of the anti-Opa SBA response for the first time. Opa cross-reactive antibodies have previously been found following immunisation of mice, and additional investigations utilising a larger panel of Opa variants is required to fully explore the occurrence of any cross-reactivity. This would ideally include study of both mouse and human sera in those who have either received Opa-containing vaccines (including OMVs) or following invasive disease or meningococcal carriage. SBA titres following immunisation with recombinant proteins were between 4- and 16-fold lower than following OMV immunisations, which may be expected given that OMVs contain a large number of antigens, including the immunodominant PorA [24], [41], [42]. This is also the likely explanation for the similarity of responses following immunisation with Opa-positive or Opa-negative OMVs, with the inclusion of Opa not conferring either advantage or disadvantage with respect to SBA response. However, given the lack of bactericidal activity demonstrated against strains not expressing the same Opa, it is likely that all of the SBA activity observed after protein immunisation was Opa-mediated, providing further support for pursuing Opa as a vaccine candidate. Importantly, the expression of Opa within the OMVs did not lead to any inhibition of the SBA response as has been found in some *in vitro* studies [34], [35], although these effects may not necessarily be apparent in mice due to differences between the human and mouse CEACAM repertoire.

Inclusion of Opa proteins in future vaccines will require good quality epidemiological data to identify common Opa variants in circulation for inclusion in the vaccine. This would ideally include additional information on cross-reactivity of the bactericidal response following immunisation and Opa PV, which would influence the potential effectiveness of any Opa-containing vaccine. The lack of Opa expression in some strains constructed in this study which retained wild-type *opa* genes highlights the challenge that PV poses in developing Opa as a vaccine candidate. This would appear to suggest that four Opa proteins are required in a vaccine to ensure coverage against a single strain, in order to circumvent the problem of PV. However, bactericidal epitopes for anti-Opa antibodies have been described in two of the four surface-exposed loops containing hypervariable (HV) regions [17], and there is limited diversity of these HV regions among *opa* genes from hyperinvasive meningococcal isolates [43]. This lends credence to the possibility of providing broad coverage with a small number of Opa variants, despite the occurrence of PV. The importance of Opa to adhesion of meningococci to epithelial cells in the nasopharynx as the precursor to invasive disease mean it is unlikely that no Opa proteins would be expressed within a population of colonising organisms and provides the additional potential that an Opa vaccine might have activity against colonisation through induction of anti-adhesion antibody. No effect of anti-Opa antibodies (from post-mouse immunisation serum or using anti-Opa mAbs) on adhesion was demonstrated in this study using encapsulated bacteria (data not shown) [44], [45], and although Opa-mediated adhesion of apparently fully encapsulated meningococci to epithelial cells has been demonstrated [46], [47], [48], such interactions occur most effectively with acapsulate organisms [49], [50], which may represent a significant proportion of meningococci in the nasopharynx [51], [52].

DNA sequence analysis following transformation of meningococci revealed that homologous recombination of exogenous DNA can occur with just 80 bp of homology in the flanking region. *N. meningitidis* is a naturally transformable bacterial species, having the ability to take up extracellular DNA efficiently, and then incorporate it into the chromosome by homologous recombination [53]. This mechanism can be manipulated *in vitro* to modify expression of specific proteins, and has been utilised recently in the development of several meningococcal vaccines to delete, over-express or modify surface components and therefore increase the immunogenicity of OMVs derived from these strains [9], [10], [11], [12]. However, the minimum requirement for sequence homology has not been clearly defined, although most studies suggest 500 bp–1,000 bp is required either side of the selectable marker to enable efficient recombination [39], [54], [55]. Successful transformation has been achieved with 280–290 bp of homology [38], [56] and studies of *N. gonorrhoeae* have reported unpublished observations that recombination can occur with as little as 100 bp of homology, and that minor modifications in the flanking sequence are permissible [57], [58]. This study provides clear evidence that only 80 bp is sufficient to enable homologous recombination to occur. Even with this relatively small amount of flanking sequence, minor differences were tolerated during the recombination process, although it is much more likely to occur if there is 100% sequence identity, as demonstrated by the higher rates of recombination at the same *opa* locus as that carried on the plasmid.

There are currently a limited number of selectable markers available for use in *Neisseria*, and this study highlights some important factors to optimise performance of these markers and cloning methods. We found that for KanR, the orientation must be the same as the target gene to obtain the resistance phenotype in *N. meningitidis* [59], although either orientation will confer kanamycin resistance in *E. coli*. For EryR or TetR, insertion can be in either orientation, which makes these markers easier to use for genetic manipulation of meningococci. Additional unpublished observations from our laboratory have shown that the concentration of kanamycin required in selective media following meningococcal transformation is strain dependent. The data also suggest that removal of a selectable marker is a feasible strategy for the manipulation of meningococcal DNA, when efficiency of transformation and recombination is high. This increases the number of genes which can be altered in a single isolate, but a non-antibiotic marker which is easily selectable when it is inserted or removed would greatly facilitate construction of mutant strains, either for the development of new vaccines or investigation of the biology of the organism. This would also ideally involve production of strains which are able to constitutively express different phase variable proteins. Construction of a strain which constitutively expresses Opa has been described for an *opa* gene of *N. gonorrhoeae* [60], but this has a number of inherent difficulties, including the presence of a repeat sequence of variable length within the open reading frame, a previous finding that high-level expression of Opa within *E. coli* was toxic to the host cells [60] and the requirement for high fidelity at each stage of DNA manipulation.

Our library of *opa*-deficient strains will allow further assessment of Opa as a potential vaccine candidate against meningococcal disease caused by all serogroups. These mutants have a potential use to further investigate the immunomodulatory effects of Opa, in order to identify the most immunogenic variants for use in a future meningococcal vaccine. In addition it will be possible to further investigate the role of Opa proteins during adhesion and invasion, including differences between Opa variants and the effects of expression of different combinations of Opa proteins on the bacterial surface. We have demonstrated that transformation of *N. meningitidis* is possible with very short regions of homology between plasmid and chromosomal DNA. Such shorter fragments of DNA should be easier to manipulate during cloning, so this finding will ease the construction of genetically modified meningococci in the attempt to develop a broadly protective meningococcal vaccine. These mutants demonstrate there is a significant and specific anti-Opa bactericidal antibody response following murine immunisation with recombinant protein, and no inhibition of the response when Opa is contained within OMVs supporting further investigation of Opa as a component of future meningococcal vaccines.

## Materials and Methods

### Ethics Statement

Animal studies were conducted according to the UK Home Office regulations and were approved by the local ethics committee at the National Institute for Biological Standards and Control (Home Office Project Licence Number 80/2157). Samples were obtained following terminal general anaesthesia and all efforts were made to minimise suffering.

### Bacterial strains and growth conditions

*Escherichia coli* was grown in Luria-Bertani (LB) media at 37°C for 16–18 hours, with shaking at 220 rpm for broth cultures. *N. meningitidis* was grown on brain heart infusion (BHI) agar (Merck, Darmstadt, Germany) supplemented with Levinthal's base (10% v/v) at 37°C in a humidified 5% CO_2_ atmosphere for 16–18 hours. Selective media was supplemented with ampicillin (100 µg/ml), kanamycin (100 µg/ml), erythromycin (300 µg/ml for *E. coli* and 5 µg/ml for *N. meningitidis*) or tetracycline (12 µg/ml for *E. coli* and 2 µg/ml for *N. meningitidis*) (Sigma-Aldrich, Gillingham, UK).

### Transformation of Escherichia coli and Neisseria meningitidis

Chemically competent *E. coli* DH5α were prepared using calcium chloride [61], [62] and transformation of *E. coli* for the propagation of plasmids was performed using standard methods [62]. Transformed cells were plated onto selective LB agar and incubated at 37°C for 16–18 hours, or 36–40 hours when selecting for erythromycin resistance. *N. meningitidis* was transformed using the spot transformation technique [57]. Briefly, 10 µl (approximately 10^8^ colony forming units [cfu]) of bacterial suspension from overnight growth was incubated with approximately 1 µg of linearised or supercoiled plasmid DNA or chromosomal DNA and plated over a 1–2 cm diameter region on BHI agar. Reactions were incubated at 37°C, 5% CO_2_ for 4–8 hours before bacteria were plated onto selective BHI agar and incubated for a further 16–18 hours (36–40 hours for the introduction of erythromycin resistance). When transforming *N. meningitidis* to remove an antibiotic resistance cassette, transformation reactions were plated onto non-selective BHI agar to achieve 500–1,000 cfu per plate before incubation. After 16–18 hours, a nitrocellulose membrane (pore size 0.45 µm) was used to transfer colonies to selective BHI agar plates. After 16–18 hours paired plates were visually screened to identify colonies which were present on non-selective media only. Colonies were re-grown on both selective and non-selective media to confirm loss of antibiotic resistance.

### Construction of meningococcal outer membrane vesicles

Bacteria were grown on BHI agar at 37°C, 5% CO_2_ for 16–18 hours. A few individual colonies were picked and resuspended in 200–1000 µl of PBS, before plating 25–50 µl of this suspension onto BHI agar. OMVs were produced from this suspension as previously described [63] with the exception that thiomersal was excluded from all buffers.

### Characterisation of bacteria and outer membrane vesicles

Bacteria and OMVs were initially characterised using anti-Opa mAbs 15-1-P5.5 and MN20E12.70 [64]. There was some cross-reactivity of the mAbs between the Opa variants found in H44/76, with 15-1-P5.5 recognising OpaA and OpaJ, and the target epitope for MN20E12.70 being present in OpaB and OpaD. Expression of other proteins was detected using anti-PorA P1.7 mAb MN14C11.6 [65], anti-PorB P3.15 mAb 2-1-P15 [65], anti-RmpM mAb 173,G-1 [66] and anti-fHbp variant 1 mAb JAR4 [67]. Antibody binding was detected using alkaline phosphatase-conjugated anti-mouse IgG and BCIP/NBT (Sigma-Aldrich). Immunodot-blotting of meningococcal cell suspensions was performed as previously described after bacteria had been fixed in 70% ethanol [21]. Total protein concentrations of OMVs were determined using a modified Lowry assay. Protein profiles were analysed by SDS-PAGE and immunoblotting was carried out using standard methods to confirm expression of Opa and other proteins [68].

### Recombinant DNA methods

Plasmid DNA was isolated from *E. coli* using the QIAprep Miniprep kit (Qiagen, Crawley, UK) before being screened by polymerase chain reaction (PCR) using the universal primers M13 forward (5′-GTAAAACGACGGCCAG-3′) and M13 reverse (5′-CAGGAAACAGCTATGAC-3′) and restriction enzyme digestion. Chromosomal DNA from *N. meningitidis* was isolated using the QIAamp DNA Mini kit (Qiagen). Following transformation, the identity of each new meningococcal transformant was confirmed by growth on selective media and analysis by PCR (table 3). PCR was carried out using primers at a concentration of 0.2 µM per reaction (Sigma-Aldrich). Each reaction contained 1.25 units Taq DNA Polymerase (Qiagen) and 200 µM each of dATP, dCTP, dGTP and dTTP in a total volume of 50 µl. PCR conditions were: initial denaturation at 95°C for 5 minutes; 30 cycles of denaturation at 95°C for 30 seconds, annealing at 55°C for 30 seconds, extension at 68°C for 3 minutes; final elongation at 68°C for 3–10 minutes. TA cloning was performed using the vector pCR2.1-TOPO (Invitrogen, Paisley, UK) according to the manufacturer's instructions. Ligation reactions were performed using standard methods [62].

**Table 3:** PCR primers used for screening *opa* genes following transformation of *Neisseria meningitidis*.

Target of PCR	Forward primer	Reverse primer	Product size (bp)				
			*opa* (wt*)	*opa::ery*	*opa::kan*	*opa::tet*	Δ*opa*
EryR	ery-bamf	ery-bamr	–	1210	–	–	–
KanR	kan-if	kan-ir	–	–	963	–	–
TetR	tetF	tetR	–	–	–	1603	–
*opaA*	nitF1SalI	NMB0444-4SacII	3510	4523	4595	5378	–
*opaB*	NMB1634-4SalI	0464 opaBrevSacII	3676	4673	4743	–	–
*opaD*	NMB1464-7SalI	NMB1466-0SacII	2703	3700	3770	4553	–
*opaJ*	acthR2SalI	pipSEQRSacII	4489	5502	5574	–	4310
*opaA::ery*	nitF1SalI	ery-bamf	–	1703	–	–	–
*opaA::kan*	nitF1SalI	kan-5-out	–	–	887	–	–
*opaB::ery*	ery-bamf	0464 opaBrevSacII	–	1950	–	–	–
*opaB::kan*	NMB1634-4SalI	kan-3-out	–	–	2881	–	–
*opaD::ery*	ery-bamr	NMB1466-0SacII	–	2459	–	–	–
*opaD::kan*	kan-3-out	NMB1466-0SacII	–	–	1407	–	–
*opaD::tet*	tetR	NMB1466-0SacII	–	–	–	2923	–
*opaJ::ery*	ery-bamr	pipSEQRSacII	–	3552	–	–	–
*opaJ::kan*	kan-3-out	pipSEQRSacII	–	–	2685	–	–
Δ*opaJ*	acthR2SalI	opaFSalI	2370	–	–	–	2191

Following each transformation, three PCR reactions were carried out at each *opa* locus to confirm recombination. The antibiotic resistance cassette was amplified using internal primers to confirm it had inserted into the genome. The antibiotic resistance cassette was amplified with one internal primer and one primer within a locus-specific gene adjacent to *opa* to confirm insertion had occurred within the target *opa* gene. Finally, the entire *opa* locus was amplified using primers in adjacent genes to confirm a double crossover event had occurred.

* wt = wild-type. Primer sequences were: kan-if, 5′-AGCCATATTCAACGGGAAAC-3′; kan-ir, 5′-TTTGCTTTGCCACGGAAC-3′; tetF, 5′-TTGATGCTCTTGATCTTCC-3′; tetR, 5′-TAACAGCAAACAGTAATGG-3′; NMB1464-7SalI, 5′-TGCAGAGTCGACGGCATCAACACCCATGC-3′; NMB1466-0SacII, 5′-CCGCCTCCGCGGTTATGTTGTGCGACCAGTCC-3′; kan-5-out, 5′-TCAAAAATATGGTATTGATAATCC-3′; kan-3-out, 5′-TGTAACATCATTGGCAACGC-3′. Other primer sequences are described in table 1.

### DNA sequencing

Dye-terminator DNA sequencing was used following amplification of *opa* genes by PCR. Amplicons were purified by precipitation with polyethylene glycol 8000 (20% w/v)/NaCl (2.5 M), followed by centrifugation at 2,750× *g* for 1 hour at 4°C and washing with ethanol (70%). Sequencing reactions contained 1.3 pmol oligonucelotide primer (Sigma-Aldrich) and 0.5 µl BigDye Terminator Ready Reaction Mix (Applied Biosystems, Paisley, UK) in a total of 10 µl. The following conditions were used: 30 cycles of 95°C for 10 seconds, 50°C for 6 seconds and 60°C for 2 minutes. Sequencing primers were O85 (5′-GGCATAATCTGCCGCTATCC-3′) [69] and OpaFwdII (5′-TATATTGTGTTGAAACATCG-3′), which was based on O3510 [20]. Extension products were purified by precipitation with ethanol (100%)/sodium acetate (115 mM, pH 4.6) and centrifugation as above. Labelled extension products were separated by capillary electrophoresis on a 3730×l DNA Analyzer (Applied Biosystems) at the Department of Zoology Sequencing Facility, University of Oxford. Sequence trace chromatograms were assembled into consensus sequences using the Staden sequence analysis package [70], using Gap4 shotgun assembly. Sequences were aligned using Clustal X (version 2.0) [71] and visualised with GeneDoc (version 2.7.000) [72]. No new sequence data were generated in this study.

### Preparation of recombinant Opa proteins

Refolded recombinant Opa proteins were produced in *E. coli* inclusion bodies from the pET22b(+)/*opa* plasmids as previously described [21], [73] with an additional purification step. Ion exchange chromatography was performed following heparin affinity chromatography using a Resource S column (GE Healthcare, Buckinghamshire, UK) equilibrated in sodium acetate (50 mM, pH 4.6) containing LDAO (0.1%). Opa protein was then eluted by application of a gradient from 0 to 1 M NaCl. Fractions containing Opa proteins were pooled, dialysed into PBS containing LDAO (0.1%) and stored at −20°C.

### Immunisation of mice

Groups of ten 6–7 week old female BALB/c mice (Charles River, Margate, UK) were immunised subcutaneously with 5 µg of recombinant Opa protein or OMVs (2.5 µg total protein) on days 0, 21 and 35. The oil-in-water emulsion Sigma Adjuvant System (Sigma-Aldrich), in a total volume of 2.4 ml of antigen solution per vial, was reconstituted with the antigen on the day of immunisation. Blood was collected by cardiac puncture on day 42 and serum separated by centrifugation at 16,100× *g* for 10 minutes.

### Serum bactericidal assay

Bactericidal activity in mouse serum pooled within each immunisation group was quantified by SBA based on a published method [74]. Briefly, pooled murine sera was heated at 56°C for 30 minutes to deactivate endogenous complement and then diluted to give a range from 1∶4 to 1∶1024. Diluted sera was incubated with mid-log phase meningococci (125 cfu) and baby rabbit complement (lot number 11330, PelFreez Biologicals, Rogers, AR) at a final concentration of 25% (v/v). Reactions were incubated for 60 minutes at 37°C in a humidified 5% CO_2_ atmosphere. A sample of each reaction was spread by tilting onto BHI agar plates, which were incubated overnight. The bactericidal antibody titre was reported as the reciprocal of the highest serum dilution at which 50% bacterial survival was observed. Each serum sample was analysed in duplicate against each target strain. When SBA titres were too high to be determined, additional assays were performed with dilution of sera to 1∶16,384.

## Supporting Information

Figure S1 **Sequence alignment of wild type** ***opaB*** **, plasmid** ***ΔopaD::ery*** **,** ***ΔopaB::ery*** **from strain M004 and** ***ΔopaB::ery*** **from strain M005.** Strains M004 and M005 were constructed by disruption of *opaB* with a plasmid based on *opaD*, containing either *ΔopaD::ery* (strain M004) or *ΔopaD::kan* (strain M005). For strain M004, homologous recombination had to occur between positions 89 (position of PCR primer used to clone *ΔopaD* into the plasmid) and 712 (*Sgr*AI site where *ermC* was inserted) - both regions are highlighted in black. Differences between the chromosomal *opaB* and plasmid *ΔopaD* (highlighted in grey) reveal that the *ΔopaB* locus of strain M004 was identical to the wild type until at least position 633, so recombination occurred between positions 633 and 712. Similarly, the *ΔopaB* locus of strain M005 was identical to the plasmid *ΔopaD* at all positions where differences existed, so recombination occurred between positions 89 and 170. Other notable features are the longer CR tract within *ΔopaD* and deletion of a 197 bp-fragment compared to wild type *opaB*.
(TIF)

Figure S2 **Characterisation of Opa-positive and Opa-negative bacteria and OMVs.** **(a) and (b) Immunodot-blot of ethanol-fixed bacteria. (c) and (d) SDS-PAGE and immunoblotting of OMVs.** (a) Immunodot-blot layout; (b) Immunodot-blotting using mAbs 15-1-P5.5 (anti-OpaA) and MN20E12.70 (anti-OpaD) confirmed expected Opa phenotype of all four strains as predicted by the DNA sequence data. 1 and 5 = H44/76; 2 and 6 = M014; 3 and 7 = M001; 4 and 8 = M002; 1–4 = blotting with mAb 15-1-P5.5; 5–8 = blotting with mAb MN20E12.70. (c) SDS-PAGE of OMVs. Lanes A and F = low-range molecular weight standards (Bio-Rad, Hemel Hempstead, UK); lane B = H44/76; lane C = M014; lane D = M001; lane E = M002. The black arrow highlights an additional band present in strains M001 and M002 only, which was confirmed as representing Opa based on immunoblotting. Profiles of all other proteins were comparable between strains (figure S3). (d) Immunoblotting confirmed expression of OpaA and OpaD in the relevant strains, as well as low level expression of Opa in the wild-type strain, and no Opa expression in the Opa-negative strain. Lanes 1–4 = blotting with mAb 15-1-P5.5; lanes 5–8 = blotting with mAb MN20E12.70. Lanes 1 and 5 = H44/76; lanes 2 and 6 = M014; lanes 3 and 7 = M001; lanes 4 and 8 = M002. H44/76 = wild-type; M014 = Opa-negative; M001 = OpaA+ OpaD+; M002 = OpaD+.
(TIF)

Figure S3 **Expression of non-Opa major meningococcal outer membrane proteins by immunoblotting of OMVs.** Immunoblotting with specific mAbs confirmed that expression of PorA, PorB, RmpM and fHbp was comparable in strains H44/76, M014, M001 and M002.
(TIF)

Table S1 **Number of coding repeat (CR) sequences in the N-terminal region of each** ***opa*** **gene in the wild-type and** ***opa*** **-deficient mutant strains constructed.** Opa protein expression should occur if the number of CR sequences is a multiple of three since the mature polypeptide is translated in-frame. Genes where Opa protein expression would be expected based on the nucleotide sequence are shown in bold. No Opa expression would be expected for other *opa* genes. Δ: *opa* gene disrupted, so protein expression not possible; *Change in number of CRs from parent strain.
(DOC)
