# Correction: Construction of Opa-Positive and Opa-Negative Strains of *Neisseria meningitidis* to Evaluate a Novel Meningococcal Vaccine

**DOI:** 10.1371/annotation/900641dd-0b06-4a77-9e2c-b7161dd49e1d

**Published:** 2013-10-01

**Authors:** Manish Sadarangani, J. Claire Hoe, Martin J. Callaghan, Claire Jones, Hannah Chan, Katherine Makepeace, Hélène Daniels-Treffandier, Mary E. Deadman, Christopher Bayliss, Ian Feavers, Peter van der Ley, Andrew J. Pollard

Original text: "Expression of other proteins was detected using anti-PorA P1.7 mAb MN14C11.6 [65], anti-PorB P3.15 mAb 2-1-P15 [65], anti-RmpM mAb 173,G-1 [66] and anti-fHbp variant 1 mAb JAR4 [67]"

Corrected text: "Expression of other proteins was detected using anti-PorA P1.7 mAb MN14C11.6 [65], anti-PorB P3.15 mAb 2-1-P15 [65], anti-RmpM mAb 173,G-1 [66] (kind gift of Jan Kolberg, Norwegian Institute of Public Health, Oslo) and anti-fHbp variant 1 mAb JAR4 [67]"

