# Supplementary figures and images for: Construction of Opa-Positive and Opa-Negative Strains of Neisseria meningitidis to Evaluate a Novel Meningococcal Vaccine
Source: PLoS One. 2012 Dec 12;7(12):e51045. doi: 10.1371/journal.pone.0051045 (PMC3521020; doi:10.1371/journal.pone.0051045)

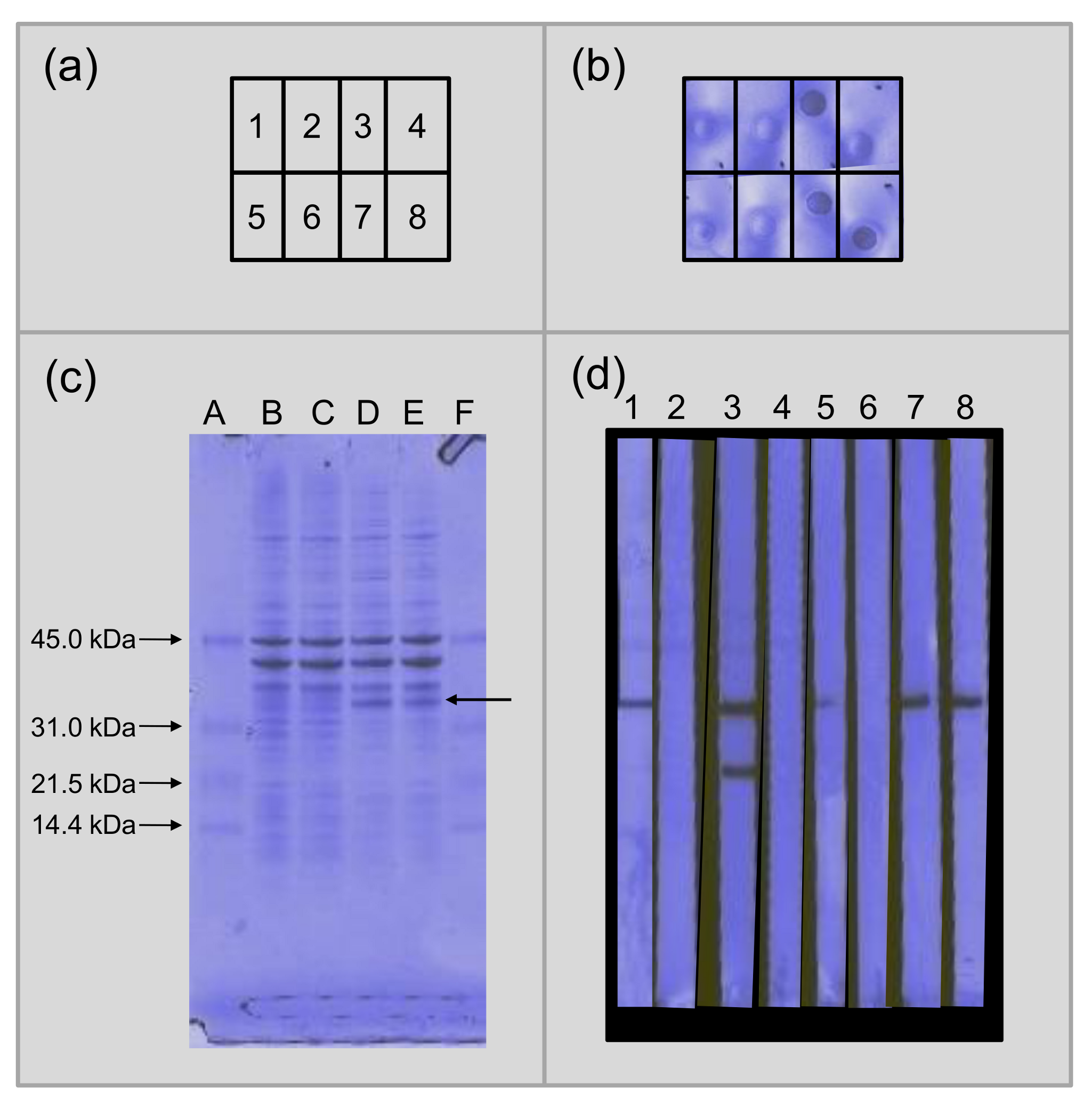

Supplement: Figure S2 — Characterisation of Opa-positive and Opa-negative bacteria and OMVs. (a) and (b) Immunodot-blot of ethanol-fixed bacteria. (c) and (d) SDS-PAGE and immunoblotting of OMVs. (a) Immunodot-blot layout; (b) Immunodot-blotting using mAbs 15-1-P5.5 (anti-OpaA) and MN20E12.70 (anti-OpaD) confirmed expected Opa phenotype of all four strains as predicted by the DNA sequence data. 1 and 5 = H44/76; 2 and 6 = M014; 3 and 7 = M001; 4 and 8 = M002; 1–4 = blotting with mAb 15-1-P5.5; 5–8 = blotting with mAb MN20E12.70. (c) SDS-PAGE of OMVs. Lanes A and F = low-range molecular weight standards (Bio-Rad, Hemel Hempstead, UK); lane B = H44/76; lane C = M014; lane D = M001; lane E = M002. The black arrow highlights an additional band present in strains M001 and M002 only, which was confirmed as representing Opa based on immunoblotting. Profiles of all other proteins were comparable between strains (figure S3). (d) Immunoblotting confirmed expression of OpaA and OpaD in the relevant strains, as well as low level expression of Opa in the wild-type strain, and no Opa expression in the Opa-negative strain. Lanes 1–4 = blotting with mAb 15-1-P5.5; lanes 5–8 = blotting with mAb MN20E12.70. Lanes 1 and 5 = H44/76; lanes 2 and 6 = M014; lanes 3 and 7 = M001; lanes 4 and 8 = M002. H44/76 = wild-type; M014 = Opa-negative; M001 = OpaA+ OpaD+; M002 = OpaD+. (TIF) [file pone.0051045.s002.tif]
