# Supplementary material for: Construction of Opa-Positive and Opa-Negative Strains of Neisseria meningitidis to Evaluate a Novel Meningococcal Vaccine
Source: PLoS One. 2012 Dec 12;7(12):e51045. doi: 10.1371/journal.pone.0051045 (PMC3521020; doi:10.1371/journal.pone.0051045)
Supplement: Table S1 — Number of coding repeat (CR) sequences in the N-terminal region of each opa gene in the wild-type and opa -deficient mutant strains constructed. Opa protein expression should occur if the number of CR sequences is a multiple of three since the mature polypeptide is translated in-frame. Genes where Opa protein expression would be expected based on the nucleotide sequence are shown in bold. No Opa expression would be expected for other opa genes. Δ: opa gene disrupted, so protein expression not possible; *Change in number of CRs from parent strain. (DOC) [file pone.0051045.s004.doc]

| **Strain** | ***opaA*** | ***opaB*** | ***opaD*** | ***opaJ*** | **Parent strain** |
| --- | --- | --- | --- | --- | --- |
| H44/76 wt | 10 | 8 | 14 | 11 | – |
| M001 | **9*** | 8 | **15*** | Δ | H44/76 wt |
| M002 | Δ | 8 | **15*** | 11 | H44/76 wt |
| M003 | 10 | 8 | Δ | 11 | H44/76 wt |
| M004 | 10 | Δ | 14 | 11 | H44/76 wt |
| M005 | Δ | Δ | **15** | 11 | M002 |
| M006 | Δ | 8 | Δ | 11 | M002 |
| M007 | Δ | 8 | **15** | Δ | M002 |
| M008 | 10 | 8 | Δ | Δ | M001 |
| M009 | 10 | Δ | 14 | Δ | M004 |
| M010 | 10 | Δ | Δ | 11 | M004 |
| M011 | 10 | Δ | 14 | Δ | M009 |
| M012 | Δ | Δ | 14 | Δ | M011 |
| M013 | 10 | Δ | Δ | Δ | M011 |
| M014 | Δ | Δ | Δ | Δ | M012 |
| M015 | Δ | Δ | Δ | Δ | M013 |
| M016 | Δ | 8 | Δ | Δ | M007 |
| M017 | Δ | 8 | Δ | Δ | M008 |
| M018 | Δ | Δ | Δ | 11 | M005 |
| M019 | Δ | Δ | Δ | 11 | M010 |

Table S1. Number of coding repeat (CR) sequences in the N-terminal region of each *opa* gene in the wild-type and *opa*-deficient mutant strains constructed.

Opa protein expression should occur if the number of CR sequences is a multiple of three since the mature polypeptide is translated in-frame. Genes where Opa protein expression would be expected based on the nucleotide sequence are shown in bold. No Opa expression would be expected for other *opa* genes.

Δ: *opa* gene disrupted, so protein expression not possible; *Change in number of CRs from parent strain.
